# Hemispheric Difference of Regional Brain Function Exists in Patients With Acute Stroke in Different Cerebral Hemispheres: A Resting-State fMRI Study

**DOI:** 10.3389/fnagi.2021.691518

**Published:** 2021-07-09

**Authors:** Jingchun Gao, Canhong Yang, Qixiong Li, Lanpin Chen, Yijing Jiang, Songyan Liu, Jing Zhang, Gang Liu, Junqi Chen

**Affiliations:** ^1^Department of Rehabilitation Medicine, Third Affiliated Hospital of Southern Medical University, Guangzhou, China; ^2^Department of Rehabilitation Medicine, Foshan Hospital of Traditional Chinese Medicine, Foshan, China; ^3^Department of Neurology, Third Affiliated Hospital of Southern Medical University, Guangzhou, China; ^4^School of Traditional Chinese Medicine, Southern Medical University, Guangzhou, China; ^5^Department of Rehabilitation Medicine, Rehabilitation Hospital, Fujian University of Traditional Chinese Medicine, Fuzhou, China; ^6^Department of Neurology, China-Japan Union Hospital of Jilin University, Changchun, China; ^7^Department of Rehabilitation Medicine, Nanfang Hospital, Southern Medical University, Guangzhou, China

**Keywords:** resting-state functional magnetic resonance imaging, AIS, amplitude of low frequency fluctuations, regional homogeneity, dominant hemisphere, non-dominant hemisphere

## Abstract

**Objective:**

To explore the different compensatory mechanisms of brain function between the patients with brain dysfunction after acute ischemic stroke (AIS) in the dominant hemisphere and the non-dominant hemisphere based on Resting-state Functional Magnetic Resonance Imaging (Rs-fMRI).

**Methods:**

In this trial, 15 healthy subjects (HS) were used as blank controls. In total, 30 hemiplegic patients with middle cerebral artery acute infarction of different dominant hemispheres were divided into the dominant hemisphere group (DH) and the non-dominant hemisphere group (NDH), scanned by a 3.0 T MRI scanner, to obtain the amplitude of low-frequency fluctuations (ALFF) and regional homogeneity (ReHo) and compare the differences.

**Results:**

Compared with the HS, increased ALFF values in the brain areas, such as the bilateral midbrain, were observed in DH. Meanwhile decreased ReHo values in the brain areas, such as the right postcentral gyrus (BA3), were also observed. Enhanced ALFF values in the brain areas, such as the left BA6, and enhanced ReHo values in the brain areas, such as the left precuneus, were observed in the NDH. The ALFF and ReHo values of the right BA9 and precentral gyrus were both increased. Compared with DH, the NDH group showed lower ALFF values in the left supplementary motor area and lower ReHo values in the right BA10.

**Conclusion:**

After acute infarction in the middle cerebral artery of the dominant hemisphere, a compensation mechanism is triggered in brain areas of the ipsilateral cortex regulating motor-related pathways, while some brain areas related to cognition, sensation, and motor in the contralateral cortex are suppressed, and the connection with the peripheral brain regions is weakened. After acute infarction in the middle cerebral artery of the non-dominant hemisphere, compensatory activation appears in motor control-related brain areas of the dominant hemisphere. After acute middle cerebral artery infarction in the dominant hemisphere, compared with the non-dominant hemisphere, functional specificity in the bilateral supplementary motor area weakens. After acute middle cerebral artery infarction in different hemispheres, there are hemispheric differences in the compensatory mechanism of brain function.

## Introduction

The brain is the most complex organ in humans, and research on it is the most advanced and popular field in life science. With the implementation of the “Brain Plan,” more researchers nowadays are exploring cerebral functional changes with an aim to study various cerebral diseases.

As one of the cerebral diseases in the “brain program,” stroke is listed as the primary cause of disability and death (Wang et al., 2013, 2017) due to its characteristics of high incidence, high disability rate, and high mortality rate. The existing basic researches mainly concentrate on proteomics, genomics, and metabolomics (Nguyen et al., 2016; Hasin et al., 2017; Wang et al., 2020). Neuroimaging technology is a research focus in the field of *in vivo* research on post-stroke injury. With the continuous development of neuroimaging technology, neuroimaging diagnosis is no longer limited to observing the changes in brain histomorphology but has entered the stage of comprehensive diagnosis by combining brain morphology with function (Hojjati et al., 2019). In particular, understanding of cerebral reorganization after the injury in the central nervous system has been increased significantly through the non-invasive examination of functional MRI.

Functional magnetic resonance imaging (fMRI) is one of the representative neuroimaging techniques and can be divided into task-state fMRI technology and resting-state fMRI technology (resting-state fMRI, rs-fMRI). Under the rest state, Rs-fMRI (Li et al., 2016; Wang et al., 2016) receives feedback on neuronal activity by detecting the change of the hemodynamics in the local brain area after the spontaneous cerebral function activity and measuring the change of deoxyhemoglobin content. It can also reflect the pathophysiological changes of cerebral functions in the resting state and directly display the location, range, and size of the activated area of cerebral functions with accurate positioning. This is beneficial to compare the rs-fMRI results of acute ischemic stroke in different lesions and is more meaningful for stroke patients with dysfunction in clinical diagnosis and treatment evaluation (Golestani et al., 2013).

At present, clinical researchers have utilized different analytical methods of rs-fMRI to study a variety of brain diseases. However, most studies only use a single parameter (ALFF/fALFF/ReHo/FC) of rs-fMRI to observe local or overall functional changes in the brain after stroke (Yin et al., 2013; van Hees et al., 2014). Moreover, functional disorders in patients with acute stroke in the dominant hemisphere (left hemisphere) differ significantly from those in the non-dominant hemisphere (right hemisphere) clinically. Physiologically, there are hemispheric differences between the dominant hemisphere and non-dominant hemisphere in neuroanatomy, physiology, neurotransmitters, and the control of sympathetic nerves (Yoon et al., 1997).

However, specific effects of dominant hemispheric and non-dominant hemispheric infarctions on brain function reorganization have not been reported in human trials, and there is a lack of rs-MRI studies on hemispheric differences in brain function after acute stroke.

Therefore, this study aims to explore the difference of brain function between dominant hemisphere and non-dominant hemisphere after acute middle cerebral artery infarction using two parameters of rs-fMRI: amplitude of low-frequency fluctuation (ALFF) and regional homogeneity (ReHo).

## Materials and Methods

### Study Design

This study was conducted as an exploratory case-control study.

### Participants

Ethical approval was obtained from the Ethics Committee of China-Japan Union Hospital of Jilin University on July 18, 2016 (No: 2016ks043). Participants aged 40–70 years old were recruited from January to December 2017 in this hospital, and written informed consent was obtained from each participant.

#### Healthy Subjects

In total, 15 healthy subjects were recruited into the normal group (HS). The inclusion criteria included the following: (1) moderate figure, regardless of gender; (2) regular diet and normal sleep, no addiction to smoking or alcohol, no tea or coffee for 24 h; (3) no head acupuncture and physiotherapy performed in the last month; (4) right handed. All of the above conditions were met. The exclusion criteria included the following: (1) having a history of stroke; (2) sensory aphasia/mixed aphasia/claustrophobia/dementia or other factors affecting communication and operation during the experiment; (3) pregnant and lactating women; (4) having metallic substances in the body (e.g., heart stents); (5) cerebral vascular pathological variation; (6) cardiovascular, renal, and liver diseases, tumors, or other diseases affecting the test results; (7) having underlying hypertension or diabetes or thyroid disease, and the recent disease control is not stable. Any of the above conditions were excluded.

#### Patients

A total of 1983 stroke patients with acute stroke were consecutively selected from the Department of Neurology. Based on the complexity and particularity of fMRI image acquisition and data statistics of stroke patients, this article estimates the sample size of the fMRI study. Referring to the systematic review (Guo et al., 2014) and statistical analyses (Desmond and Glover, 2002) on estimating sample size in functional MRI, and considering the 20% shedding rate, 15 patients were included in the dominant hemisphere group (DH) and the non-dominant hemisphere group (NDH), respectively. The inclusion criteria included the following: (1) meet the diagnostic criteria of Chinese Guidelines for Diagnosis and Treatment of Acute Ischemic Stroke, 2014 by the Neurology Branch of Chinese Medical Association and the Cerebrovascular Disease Group of the Neurology Branch of Chinese Medical Association; (2) first-ever ischemic stroke within 72 h after symptoms appear; (3) limb motor and sensory deficits; (4) stroke lesions located within the right or left middle cerebral artery (MCA) territory, as verified by magnetic resonance imaging (MRI) or computed tomography (CT); (5) in stable condition; (6) normal diet and sleep and no addiction to smoking, alcohol, tea, or coffee; (7) right handed. All of the above conditions were met. The exclusion criteria included: (1) hemorrhagic stroke, as verified by CT; (2) sensory aphasia/mixed aphasia/claustrophobia/dementia or other factors affecting communication and operation during the experiment; (3) pregnant and lactating women; (4) having metallic substances in the body (e.g., heart stents); (5) cerebral vascular pathological variation; (6) cardiovascular, renal and liver diseases, tumors, or other diseases affecting the test results; (7) having underlying hypertension or diabetes or thyroid disease, and the recent disease control is not stable. Any of the above conditions were excluded.

### MRI Data Acquisition

The MRI system (Siemens 3.0T, Siemens Healthineers, Germany) and the standard head coil were used to obtain data of T1MPRAGE and rs-fMRI (EPI sequence). The technician asked the participants to lie on the MRI scanning bed and fixed their heads in the coil with foam pads to keep their heads still. Participants were required to remain awake, maintain calm breathing, cover their eyes with a black eye mask, plug their ears with sponge earplugs, and try not to engage in specific thinking activities during the scanning.

T1MPRAGE scanning parameters were as follows: three D TFE sequence cross-section scans, and a high-resolution anatomical image T1WI of the whole brain is obtained. Scanning parameters were repetition, time/echo time ratio = 2300 ms/2.45 ms, flip angle = 8°, field of view (FOV) = 250 mm × 250 mm, slice thickness = 1 mm, Voxel = 1.0 mm × 1.0 mm, Matrix = 256 × 256, number of slices = 192.

Functional magnetic resonance imaging-BOLD scanning parameters were as follows: the single excitation echo plane imaging (EPI) technique was used for horizontal axis scanning, and Pulse time (TR) = 2000 ms, echo time (TE) = 30 ms, flip Angle = 90°, slice thickness = 3.5 mm, slice spacing = 0.7 mm, voxel = 3.5 mm × 3.5 mm × 3.5 mm, field of view (FOV) = 224 cm × 224 mm, phases per location = 240, matrix = 64 × 64, and number of slices = 37, covering a total of 8 min.

### Data Processing

Amplitude of low-frequency fluctuations and ReHo values of the three groups were calculated, respectively, based on the Matlab 2012a platform and by using the DPABI toolkit to launch statistical parameter maps (SPM 12) after removing time point, time correction, head movement correction, registration, de-linear drift, covariate removal, and image filtering, etc. Then, based on the two independent samples *t* test in the rest 1.8 software package, the three sets of data were compared and analyzed to get statistical parameters maps. We identified and corrected (AlphaSim correction, Cluster Size 27, rmm = 4, *P* < 0.005) the statistical parameter maps to achieve the anatomical location and activation intensity of brain regions with significant changes in ALFF and ReHo. The maps were finally calibrated by an experienced neurologist with anatomical knowledge and clinical experience. When DH or NDH was compared with HS, sex, age, and head movements were used as covariates; when DH and the NDH were compared, sex, age, course of disease, systolic blood pressure, diastolic blood pressure, and NHISS score were used as covariates.

In addition, data such as gender were measured by χ2 test and other data like age were checked by *t*-test. Statistical analysis was completed with statistical software SPSS20.0.

## Results

### General Information

**FIGURE 1:**
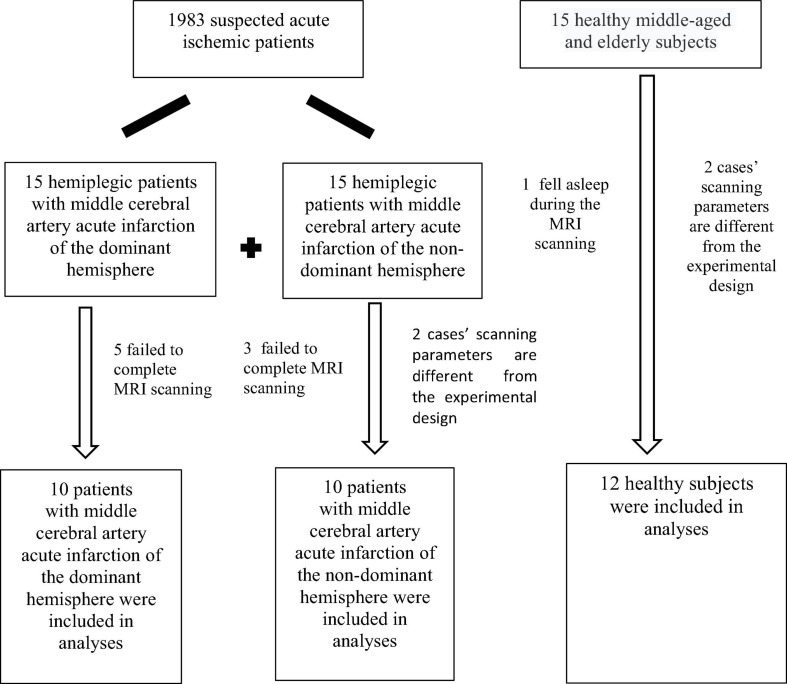
Integration of stroke patients and healthy subjects.

According to the diagnostic inclusion and exclusion criteria, 30 patients were selected into patient groups from 1983 patients suspected of acute ischemic stroke. A total of five cases in DH and three in NDH failed to complete fMRI-BOLD data collection as participants were unable to withstand long magnetic resonance scans. The EPI scanning parameters of two cases in NDH were different from the experimental design and the data were thus stripped out. Participants in one case of the normal group fell asleep during the test, and two cases had different T1 MPRAGE scanning parameters from the experimental design, and the data were all removed. A flowchart of participants is shown in Figure 1.

There were no significant differences among the three groups in their gender, course of disease, systolic and diastolic blood pressure, and NHISS scores (*P* > 0.05) (Table 1). However, the age difference of the included subjects in the three groups was statistically significant (*P* < 0.05) (Table 1).

**TABLE 1:** Demographic and clinical characteristics of all patients in three groups.

**Group**	**Case (n)**	**Gender (case)**		**Age (year)**	**Course of disease**	**Systolic pressure (mmHg)**	**Diastolic pressure (mmHg)**	**NHISS**
		**Male**	**Female**					
HS	12	4	8	56.17 ± 3.83	\	\	\	\
DH	10	7	3	63.70 ± 6.00	2.15 ± 0.94	147.80 ± 13.14	84.90 ± 10.07	5.30 ± 4.99
NDH	10	8	2	59.40 ± 7.65	2.10 ± 0.88	152.90 ± 12.34	90.40 ± 10.74	3.80 ± 1.03
Statistics		χ^2^ = 5.728		*F* = 4.437	*t* = 0.123	*t* = −0.895	*t* = −1.181	*t* = 0.931
*P* Value		0.057		0.021	0.904	0.383	0.253	0.374

*Values presented are mean ± SD (range) or median [IQR]. NIHSS, National Institutes of Health Stroke Scale.*

### fMRI Results

#### Normal Group vs. Dominant Hemisphere Group

Compared with HS, DH showed significantly increased ALFF values in the right midbrain and lobus anterior cerebelli, extending to the vermis (including cerebellar lingual), and in the left midbrain and mammillary body.

**FIGURE 2:**
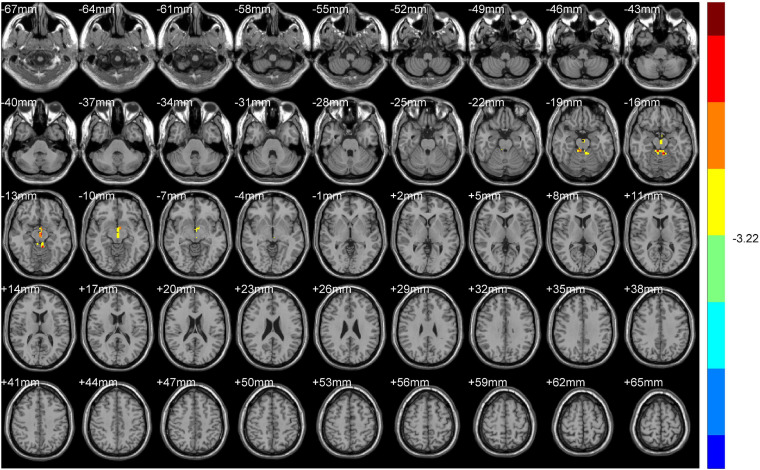
ALFF (Dominant hemisphere vs. Normal).

**FIGURE 3:**
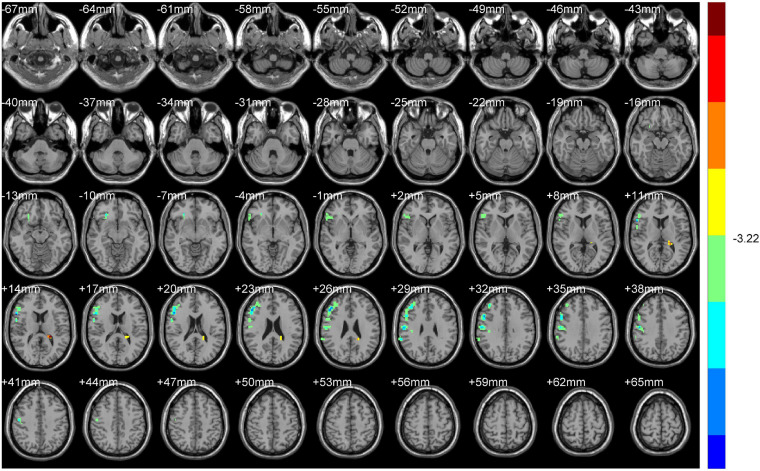
ReHo (Dominant hemisphere vs. Normal).

Increased ReHo values appeared mainly in the left caudate tai, extending to the pulvinar in DH. On the contrary, the ReHo values decreased mainly in right inferior orbital gyrus (including BA47), triangular inferior frontal gyrus (BA45), and the supra marginal and postcentral gyrus (mostly in BA3), extending to the precentral gyrus (BA4) (Table 2 and Figures 2, 3).

**TABLE 2:** Regions of DH showing significant changes in ALFF and ReHo values compared with HS.

**Parameter**	**Effect**	**Brain region**	**MNI coordinate**			**Intensity (*T*-value)**
			***X***	***Y***	***Z***	
ALFF	Enhanced	Right midbrain, lobus anterior cerebelli and Vermis	9	−33	−18	5.6226
	Enhanced	Left Midbrain and Mammillary Body	−3	−12	−12	5.0006
ReHo	Enhanced	Left Caudate Tai and Pulvinar	−21	−36	15	5.3222
	Reduced	Right inferior orbital gyrus (including BA47)	27	33	−9	–5.1493
	Reduced	Right triangular inferior frontal gyrus (BA45)	48	30	24	–7.3696
	Reduced	Right supramarginal gyrus	66	−42	30	–4.876
	Reduced	Right postcentral gyrus (mostly in BA3) and precentral gyrus (BA4)	60	−15	30	–6.2719

*X, Y, Z represent the brain space position axis, respectively, in the MiNi standardized spatial coordinate system in the left and right, front and back, and up and down position of the coordinate position.*

#### Normal Group vs. Non-dominant Hemisphere Group

Compared with HS, ALFF values of NDH increased significantly mainly in the left inferior orbital gyrus (including BA47), extending to left BA25, mainly in the left cerebellum anterior lobe (including cerebellar lingual) and Vermis, in left lentiform nuclei and globus pallidus, in the medial dorsal nucleus, extending to left midbrain, in the right caudate head, and in left BA6 and the paracentral lobule. On the contrary, the ALFF values decreased in the right BA9 and precentral gyrus.

ReHo values were increased mainly in the left Precuneus (BA7) and rectal gyrus, extending to the orbital gyrus (including BA11), mainly in the left parahippocampal gyrus, extending to BA47.

**FIGURE 4:**
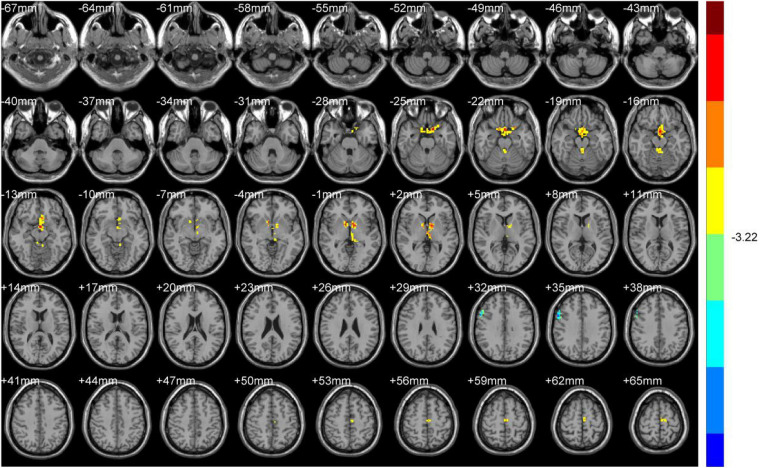
ALFF (Non-dominant hemisphere vs. Normal).

**FIGURE 5:**
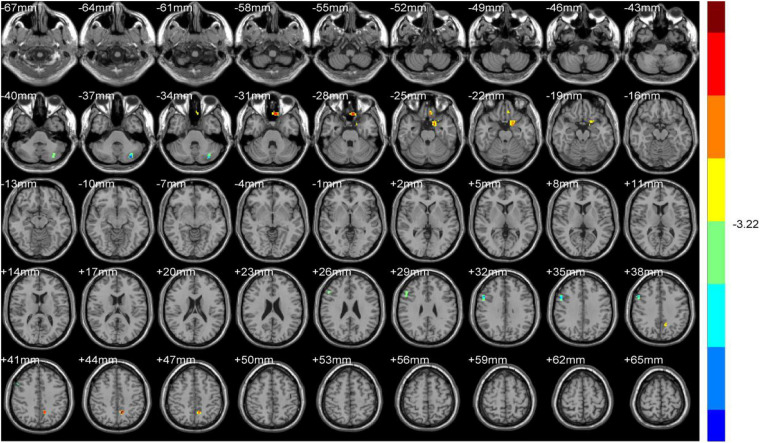
ReHo (Non-dominant hemisphere vs. Normal).

In contrast, the ReHo values decreased mainly in the left cerebellum posterior lobe, tubera valvulae, right BA9, and precentral gyrus (Table 3 and Figures 4, 5).

**TABLE 3:** Regions of NDH showing significant changes in ALFF and ReHo values compared with HS.

**Parameter**	**Effect**	**Brain region**	**MNI coordinate**			**Intensity (*T*-value)**
			***X***	***Y***	***Z***	
ALFF	Enhanced	Left inferior orbital gyrus (including BA47), BA25	−15	12	−24	6.8358
	Enhanced	Left cerebellum anterior lobe (including cerebellar lingual) and Vermis	0	−39	−28	4.633
	Enhanced	Left lentiform nuclei and globus pallidus	−9	3	3	7.4651
	Enhanced	Left medial dorsal nucleus and midbrain	−3	−12	3	5.3041
	Enhanced	Right caudate head	9	6	0	6.6047
	Enhanced	Left BA6 and paracentral lobule	−3	−15	66	5.0251
	Reduced	Right BA9 and precentral gyrus	57	9	36	–4.9502
ReHo	Enhanced	Left precuneus (BA7)	−9	−54	45	5.1723
	Enhanced	Left rectal gyrus and orbital gyrus (including BA11)	−6	33	−27	4.5542
	Enhanced	Left parahippocampal gyrus and BA47	−15	6	−24	4.386
	Reduced	Left cerebellum posterior lobe and tubera valvulae	−30	−75	−36	–5.4037
	Reduced	Right BA9 and precentral gyrus	54	12	36	–5.0544

*X, Y, Z represent the brain space position axis, respectively, in the MiNi standardized spatial coordinate system in the left and right, front and back, and up and down position of the coordinate position.*

#### Non-dominant Hemisphere Group vs. Dominant Hemisphere Group

**FIGURE 6:**
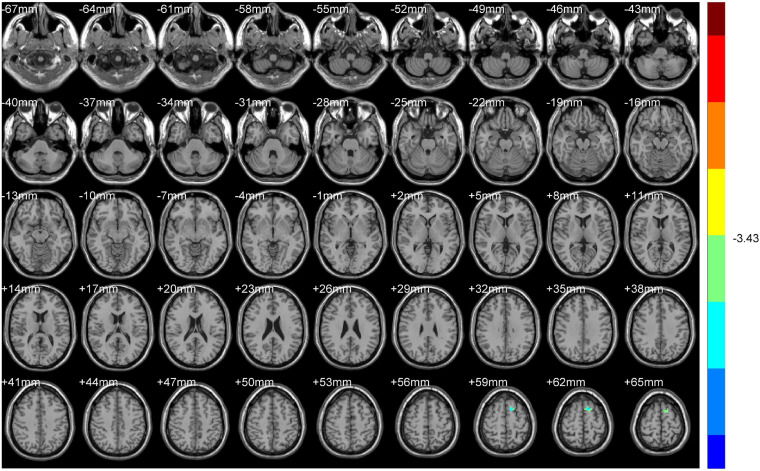
ALFF (Dominant hemisphere vs. Non-dominant hemisphere).

**FIGURE 7:**
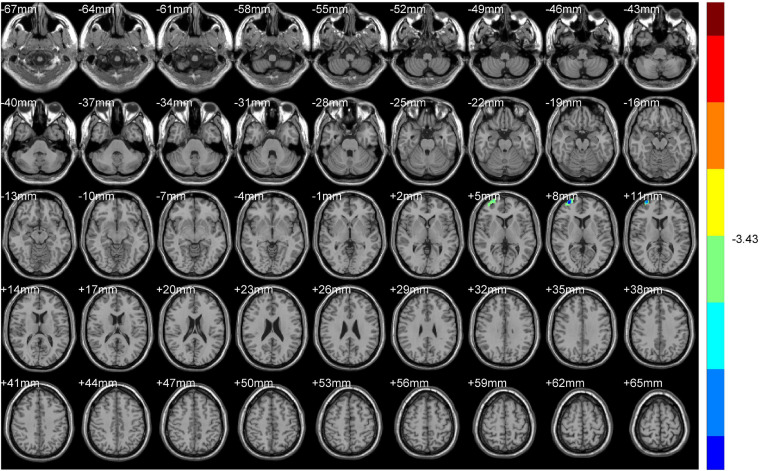
ReHo (Dominant hemisphere vs. Non-dominant hemisphere).

Compared with NDH, DH indicated significantly decreased ALFF values mainly in the left supplementary motor area (including BA6). While decreased ReHo values appeared mainly in the right BA10 (Table 4 and Figures 6, 7).

**TABLE 4:** Regions of DH showing significant changes in ALFF and ReHo values compared with NDH.

**Parameter**	**Effect**	**Brain region**	**MNI coordinate**			**Intensity (*T*-value)**
			***X***	***Y***	***Z***	
ALFF	Reduced	Left supplementary motor area (including BA6)	−12	15	63	−4.9172
ReHo	Reduced	Right BA10	30	69	9	−5.6143

*X, Y, Z represent the brain space position axis, respectively, in the MiNi standardized spatial coordinate system in the left and right, front and back, and up and down position of the coordinate position.*

## Discussion

In this study, changes in brain function in patients after acute stroke were observed, and differences between the dominant hemisphere and the non-dominant hemisphere were explored through ReHo and ALFF. For now, this is the first rs-fMRI study to compare the hemispheric differences of brain functions after stroke, especially in the acute period. Analyses of these differences are demonstrated as follows.

### Normal Group (HS) vs. Dominant Hemisphere Group (DH)

Compared with HS, the ALFF values of changed brain regions in DH were mainly activated, which means the focal neuronal activities of these regions were enhanced.

The midbrain, as the reflex center of vision and hearing, participates in the information feedback link of the closed-loop control system and can modify an executing movement in time. This is part of the typical theory of motion control (Faul et al., 2020). The cerebellar vermis (including cerebellar lingual) belongs to the anterior cerebellum and plays an important role in the transmission and feedback of the brain-cerebellar sensorimotor network (Cano-de-la-Cuerda et al., 2015; Zuk and Bertrand, 2019). The mammillary body related to the operation of emotions is a part of the limbic system of the brain. According to some literature (Habas et al., 2009), fierce emotions can affect the control of movement and produce significant behavioral responses to posture patterns or motor strategies.

Both the ALFF and ReHo values were enhanced in the left caudate tai and pulvinar. The pulvinar receives fibers from the inner and outer geniculate body and participates in the visual and auditory pathways (Tamietto et al., 2009, 2012; Pessoa and Adolphs, 2010; Faivre et al., 2012; Gainotti, 2012). The caudate tail is one of the important components of the extravertebral motor pathway, which engages in the generation and regulation of motor planning.

As is well known, when the brain receives a specific action command, a signal sent by the cerebral cortex will pass through the outgoing fiber to the skeletal muscle motor endplate and finally complete the action. Motor planning generation, coordinated control of the action, and the feedback for correction are assisted by the cortex, basal ganglia, cerebellum, and midbrain. Therefore, it is speculated that due to the infarction of the middle cerebral artery in the dominant hemisphere, it is difficult for the motor cortex to send out accurate action task signals in contrast to healthy subjects. The signal and function of the brain areas involved in the regulation of movement are strengthened as compensation to ensure the integrity and accuracy of movement.

In addition, ReHo values of the right inferior orbital gyrus (including BA47), triangular inferior frontal gyrus (including BA45), postcentral gyrus (mostly BA3), central anterior gyrus (BA4), and supramarginal gyrus decreased. These brain areas are distributed in the blood supply area of the middle cerebral artery. Vincent has shown that there is a high correlation between bilateral hemispheric ipsilateral brain interval neural activity (Vincent et al., 2007). Therefore, after an acute stroke in the dominant hemisphere, the connection between the homologous brain regions of the non-dominant hemisphere and the peripheral brain regions weakens. Among these brain regions, BA45 is responsible for performing semantic tasks and text production (Jacot-Descombes et al., 2012), BA47 is related to grammar processing of language (Levitin and Menon, 2003), the precentral gyrus (BA4) can control behavior and movement (Levitin and Menon, 2003; Jacot-Descombes et al., 2012; Itabashi et al., 2016) the postcentral gyrus (BA3) manages somatosensory, and the upper marginal gyrus (BA40) is relevant with fine movement (Hämäläinen et al., 2002). ALFF and ReHo values of some brain areas were not reduced in the dominant hemisphere, which may be related to the ischemic stroke classification (TOAST) and the compensation mechanism of cerebral collateral circulation (Liebeskind, 2003).

### Normal Group vs. Non-dominant Hemisphere Group

Compared with HS, neuronal activities of the injured side and precentral gyrus in the non-dominant hemisphere weakened in the NDH. The connection with peripheral brain regions was also reduced, which is consistent with the physiological changes caused by the responsible lesions. The precentral gyrus is the advanced motor control center, and BA9, BA6, and BA8 constitute the supplementary motor area. They serve as the main brain regions for motor sequence management and participate in the learning, planning, preparation, starting, and production of movement (Kwan et al., 1978; Meier et al., 2008). Negative activation of these two brain regions matches the hemiplegic symptoms of patients.

Interestingly, brain regions with activated ALFF and ReHo values were mainly located in the dominant hemisphere. We believe this is due to the compensation of the uninjured hemisphere (Zaaimi et al., 2012). The paracentral lobule and the precentral gyrus constitute the primary motor cortex and participate in the stage of motor execution. BA6 belongs to part of the premotor cortex and mainly engages in the initial stage of motor preparation and planning (Kwan et al., 1978; Meier et al., 2008). The supramarginal gyrus is related to the fine motor (Hämäläinen et al., 2002). Thalamus and lentiform nuclei are basal ganglia nuclei and play an important role in regulating complex and voluntary movement (Groenewegen, 2003). The lentiform nucleus, dorsomedial nucleus of the left thalamus, and the midbrain can control and purposefully perform active movement through two pathways: cortex-striatum (globus pallidus)-dorsal thalamus cortex and striatum midbrain (substantia nigra)-striatum. The precuneus are brain regions related to the cerebrum’s cognitive function (Cavanna and Trimble, 2006), which can analyze external stimuli and adjust the movement. As part of the cerebellum, the anterior lobe and superior vermis (including lingula of the cerebellum) can coordinate the motor through the feedback path of cerebellum-thalamus-(pre) motor cortex. On the other hand, the parahippocampal gyrus and cingulate cortex in BA25 are components of the limbic system and can make the cerebral cortex form higher cognitive connection and program movement to achieve an ideal motor control effect (Geyer et al., 1996). According to the theory of motor control, sensation, cognition, and activity act together in the process of motor control. Based on the theory of neuroplasticity, activation of the upper brain areas indicates a new motor control network has established in the uninjured hemisphere soon after a stroke, which can strengthen body movement coordination in many aspects and ensure active movement to the greatest extent.

In addition, the orbital gyrus (including BA47), the orbital part of the left inferior frontal gyrus, and the rectal gyrus (including BA11) were activated (Berlin et al., 2004; Camille et al., 2004; Kringelbach and Rolls, 2004; Rolls, 2004). Related to the emotion control of humans, these are components of the orbitofrontal cortex and regulate motor together with brain regions of the motor network mentioned above.

### Dominant Hemisphere Group vs. Non-dominant Hemisphere Group

Compared with the NDH, focal neuronal activities in BA6, the area responsible for motor guidance and sequence control, were lessened in the left supplementary motor area in DH. BA10 is the brain area related to emotion control movement. After Stroke, the connection between BA10 and its peripheral brain areas also reduced at the uninjured-side hemisphere. This may be related to the specific influence on the functional impairment of the brain regions responsible for the sequential control of motor guidance in the motor brain network after the injury of the dominant hemisphere (Russo et al., 2020).

### Hemispheric Differences in Stroke Can Conduct the Clinical Application of Transcranial Direct Current Stimulation

Early studies have found that when the cathode of tDCS (transcranial direct current stimulation) is close to the cell body or dendrite of nerve cells, the resting potential threshold increases and the discharge of neurons decreases, while the anode reduces the threshold of resting potential and increases the discharge of neurons (Yavari et al., 2018). Therefore, tDCS can regulate cortical excitability and has the function of nerve regulation. We believe that exploring the changes in brain function after acute stroke in different hemispheres is of great clinical significance for conducting the application of tDCS in the early phase. The cathode can be placed in the abnormal activation enhanced brain area to inhibit the local neuronal activity, while for the brain area with reduced function, the anode should be placed in the corresponding position to enhance the excitability of the neurons.

### Limitations of the Study

This study also has some limitations. First of all, due to the high requirement of resting-state functional magnetic resonance imaging on patients, although the sample size of this study was estimated according to the literature, there was still a large drop-off rate (33.3%), resulting in the study’s sample size of only 10–12 cases. But the volunteers of this study were patients with acute stroke in the middle cerebral artery, which made the recruitment difficult. Additionally, the sample size of the same type of fMRI study in stroke patients was around 10–15 cases (Guo et al., 2014). Therefore, it can be considered that the sample size of this study is sufficient to illustrate the conclusion. Secondly, this study only used ALFF and ReHo, the focal indicators of the rs-fMRI, to observe the changes in brain function of patients after acute stroke. And the results were not further discussed by the association between cerebral hemispheres after acute stroke, which needs to be verified in animal experiments.

## Conclusion

The findings of this study are as follows. Firstly, after acute infarction in the middle cerebral artery of the dominant hemisphere, a compensation mechanism is triggered in brain areas of the ipsilateral cortex regulating motor-related pathways, while some brain areas related to cognition, sensation, and motor in the contralateral cortex are suppressed, and the connection with the peripheral brain regions is weakened. Secondly, after acute infarction in the middle cerebral artery of the non-dominant hemisphere, compensatory activation appears in motor control-related brain areas of the dominant hemisphere. Thirdly, after acute middle cerebral artery infarction in the dominant hemisphere, compared with the non-dominant hemisphere, functional specificity in the bilateral supplementary motor area weakens. After acute middle cerebral artery infarction in different hemispheres, there are hemispheric differences in the compensatory mechanism of brain function.

## Data Availability Statement

The original contributions presented in the study are included in the article/supplementary material, further inquiries can be directed to the corresponding authors.

## Ethics Statement

The studies involving human participants were reviewed and approved by the Ethics Committee of the China-Japan Union Hospital at Jilin University approval. The patients/participants provided their written informed consent to participate in this study. Written informed consent was obtained from the individual(s) for the publication of any potentially identifiable images or data included in this article.

## Author Contributions

JQC was the project holder. JQC and GL contributed to conception and study design. JCG, CHY, and QXL were responsible for study follow-up and contributed to this article are tied for first place. YJJ were responsible for fcMRI acquisition. LPC and YJJ analyzed the data. SYL and JZ were responsible for patients’ recruitment, diagnosis, and treatment. JCG wrote the manuscript. CHY and QXL revised the manuscript. All authors approved the final version of the manuscript.

## Conflict of Interest

The authors declare that the research was conducted in the absence of any commercial or financial relationships that could be construed as a potential conflict of interest.

## Abbreviations

ALFF: amplitude of low-frequency fluctuations
ReHo: regional homogeneity
AIS: acute ischemic stroke
MRI: Magnetic Resonance Imaging
Rs-fMRI: Resting-state Functional Magnetic Resonance Imaging
BA: Brodmann area
fALFF: fractional amplitude of low frequency fluctuations
FC: Functional Connectivity
HS: healthy subjects
DH: the dominant hemisphere group
NDH: the non-dominant hemisphere group
NHISS: National Institute of Health stroke scale
tDCS: transcranial direct current stimulation

## References

[B1] Berlin H. A. Rolls E. T. Kischka U. (2004). Impulsivity, time perception, emotion and reinforcement sensitivity in patients with orbitofrontal cortex lesions. *Brain* 127(Pt 5) 1108–1126. 10.1093/brain/awh135 14985269

[B2] Camille N. Coricelli G. Sallet J. Pradat-Diehl P. Duhamel J. R. Sirigu A. (2004). The involvement of the orbitofrontal cortex in the experience of regret. *Science* 304 1167–1170. 10.1126/science.1094550 15155951

[B3] Cano-de-la-Cuerda R. Molero-Sánchez A. Carratalá-Tejada M. Alguacil-Diego I. M. Molina-Rueda F. Miangolarra-Page J. C. (2015). Theories and control models and motor learning: clinical applications in neuro-rehabilitation. *Neurologia* 30 32–41. 22341985 10.1016/j.nrl.2011.12.010

[B4] Cavanna A. E. Trimble M. R. (2006). The precuneus: a review of its functional anatomy and behavioural correlates. *Brain* 129(Pt 3) 564–583. 10.1093/brain/awl004 16399806

[B5] Desmond J. E. Glover G. H. (2002). Estimating sample size in functional MRI (fMRI) neuroimaging studies: statistical power analyses. *J. Neurosci. Methods* 118 115–128. 10.1016/s0165-0270(02)00121-8 12204303

[B6] Faivre N. Charron S. Roux P. Lehéricy S. Kouider S. (2012). Nonconscious emotional processing involves distinct neural pathways for pictures and videos. *Neuropsychologia* 50 3736–3744. 10.1016/j.neuropsychologia.2012.10.025 23137946

[B7] Faul L. Knight L. K. Espay A. J. Depue B. E. LaFaver K. (2020). Neural activity in functional movement disorders after inpatient rehabilitation. *Psychiatry Res. Neuroimaging* 303:111125. 10.1016/j.pscychresns.2020.111125 32585576

[B8] Gainotti G. (2012). Unconscious processing of emotions and the right hemisphere. *Neuropsychologia* 50 205–218. 10.1016/j.neuropsychologia.2011.12.005 22197572

[B9] Geyer S. Ledberg A. Schleicher A. Kinomura S. Schormann T. Bürgel U. (1996). Two different areas within the primary motor cortex of man. *Nature* 382 805–807. 10.1038/382805a0 8752272

[B10] Golestani A. M. Tymchuk S. Demchuk A. Goodyear B. G. Vision-2 Study Group (2013). Longitudinal evaluation of resting-state FMRI after acute stroke with hemiparesis. *Neurorehabil. Neural Repair* 27 153–163. 10.1177/1545968312457827 22995440

[B11] Groenewegen H. J. (2003). The basal ganglia and motor control. *Neural Plast.* 10:108384. 10.1155/NP.2003.107 PMC2565420 14640312

[B12] Guo Q. Thabane L. Hall G. McKinnon M. Goeree R. Pullenayegum E. (2014). A systematic review of the reporting of sample size calculations and corresponding data components in observational functional magnetic resonance imaging studies. *Neuroimage* 86 172–181. 10.1016/j.neuroimage.2013.08.012 23954487

[B13] Habas C. Kamdar N. Nguyen D. Prater K. Beckmann C. F. Menon V. (2009). Distinct cerebellar contributions to intrinsic connectivity networks. *J. Neurosci.* 29:8586. 10.1523/jneurosci.1868-09.2009 19571149 PMC2742620

[B14] Hämäläinen H. Hiltunen J. Titievskaja I. (2002). Activation of somatosensory cortical areas varies with attentional state: an fMRI study. *Behav. Brain Res.* 135 159–165. 10.1016/s0166-4328(02)00145-6 12356446

[B15] Hasin Y. Seldin M. Lusis A. (2017). Multi-omics approaches to disease. *Genome Biol.* 18:83. 10.1186/s13059-017-1215-1 PMC5418815 28476144

[B16] Hojjati S. H. Ebrahimzadeh A. Babajani-Feremi A. (2019). Identification of the early stage of alzheimer’s disease using structural mri and resting-state fMRI. *Front. Neurol.* 10:904. 10.3389/fneur.2019.00904 31543860 PMC6730495

[B17] Itabashi R. Nishio Y. Kataoka Y. Yazawa Y. Furui E. Matsuda M. (2016). Damage to the left precentral gyrus is associated with apraxia of speech in acute stroke. *Stroke* 47 31–36. 10.1161/strokeaha.115.010402 26645260

[B18] Jacot-Descombes S. Uppal N. Wicinski B. Santos M. Schmeidler J. Giannakopoulos P. (2012). Decreased pyramidal neuron size in Brodmann areas 44 and 45 in patients with autism. *Acta Neuropathol.* 124 67–79. 10.1007/s00401-012-0976-6 22467063

[B19] Kringelbach M. L. Rolls E. T. (2004). The functional neuroanatomy of the human orbitofrontal cortex: evidence from neuroimaging and neuropsychology. *Prog. Neurobiol.* 72 341–372. 10.1016/j.pneurobio.2004.03.006 15157726

[B20] Kwan H. C. MacKay W. A. Murphy J. T. Wong Y. C. (1978). Spatial organization of precentral cortex in awake primates. II. motor outputs. *J. Neurophysiol.* 41 1120–1131. 10.1152/jn.1978.41.5.1120 100584

[B21] Levitin D. J. Menon V. (2003). Musical structure is processed in “language” areas of the brain: a possible role for Brodmann area 47 in temporal coherence. *Neuroimage* 20 2142–2152. 10.1016/j.neuroimage.2003.08.016 14683718

[B22] Li J. Zhang X. W. Zuo Z. T. Lu J. Meng C. L. Fang H. Y. (2016). Cerebral functional reorganization in ischemic stroke after repetitive transcranial magnetic stimulation: an fmri study. *CNS Neurosci. Ther.* 22 952–960. 10.1111/cns.12593 27421949 PMC6492855

[B23] Liebeskind D. S. (2003). Collateral circulation. *Stroke* 34 2279–2284. 12881609 10.1161/01.STR.0000086465.41263.06

[B24] Meier J. D. Aflalo T. N. Kastner S. Graziano M. S. (2008). Complex organization of human primary motor cortex: a high-resolution fMRI study. *J. Neurophysiol.* 100 1800–1812. 10.1152/jn.90531.2008 18684903 PMC2576195

[B25] Nguyen V. A. Carey L. M. Giummarra L. Faou P. Cooke I. Howells D. W. (2016). A pathway proteomic profile of ischemic stroke survivors reveals innate immune dysfunction in association with mild symptoms of depression - a pilot study. *Front. Neurol.* 7:85. 10.3389/fneur.2016.00085 27379006 PMC4907034

[B26] Pessoa L. Adolphs R. (2010). Emotion processing and the amygdala: from a ‘low road’ to ‘many roads’ of evaluating biological significance. *Nat. Rev. Neurosci.* 11 773–783. 10.1038/nrn2920 20959860 PMC3025529

[B27] Rolls E. T. (2004). The functions of the orbitofrontal cortex. *Brain Cogn.* 55 11–29. 10.1016/S0278-2626(03)00277-X 15134840

[B28] Russo A. A. Khajeh R. Bittner S. R. Perkins S. M. Cunningham Laurence J. P. Abbott F. (2020). Neural trajectories in the supplementary motor area and motor cortex exhibit distinct geometries, compatible with different classes of computation. *Neuron* 107 745–758. 10.1016/j.neuron.2020.05.020 32516573 PMC9395139

[B29] Tamietto M. Castelli L. Vighetti S. Perozzo P. Geminiani G. Weiskrantz L. (2009). Unseen facial and bodily expressions trigger fast emotional reactions. *Proc. Natl. Acad. Sci. U.S.A.* 106 17661–17666. 10.1073/pnas.0908994106 19805044 PMC2764895

[B30] Tamietto M. Pullens P. de Gelder B. Weiskrantz L. Goebel R. (2012). Subcortical connections to human amygdala and changes following destruction of the visual cortex. *Curr. Biol.* 22 1449–1455. 10.1016/j.cub.2012.06.006 22748315

[B31] van Hees S. McMahon K. Angwin A. de Zubicaray G. Read S. Copland D. A. (2014). A functional MRI study of the relationship between naming treatment outcomes and resting state functional connectivity in post−stroke aphasia. *Hum. Brain Mapp.* 35 3919–3931. 10.1002/hbm.22448 24453137 PMC6869730

[B32] Vincent J. L. Patel G. H. Fox M. D. Snyder A. Z. Baker J. T. Van Essen D. C. (2007). Intrinsic functional architecture in the anaesthetized monkey brain. *Nature* 447 83–86. 10.1038/nature05758 17476267

[B33] Wang D. Liu J. Liu M. Lu C. Brainin M. Zhang J. (2017). Patterns of stroke between university hospitals and nonuniversity hospitals in mainland china: prospective multicenter hospital-based registry study. *World Neurosurg.* 98 258–265. 10.1016/j.wneu.2016.11.006 27838433

[B34] Wang M. Gui X. Wu L. Tian S. Wang H. Xie L. (2020). Amino acid metabolism, lipid metabolism, and oxidative stress are associated with post-stroke depression: a metabonomics study. *BMC Neurol.* 20:250. 10.1186/s12883-020-01780-7 32563250 PMC7305607

[B35] Wang Y. Zheng Y. Qu S. Zhang J. Zhong Z. Zhang J. (2016). Cerebral targeting of acupuncture at combined acupoints in treating essential hypertension: an Rs-fMRI study and curative effect evidence. *Evid. Based Complement. Alternat. Med.* 2016:5392954. 10.1155/2016/5392954 PMC5149687 28003850

[B36] Wang Z. Li J. Wang C. Yao X. Zhao X. Wang Y. (2013). Gender differences in 1-year clinical characteristics and outcomes after stroke: results from the China National Stroke Registry. *PLoS One* 8:e56459. 10.1371/journal.pone.0056459 23418571 PMC3572058

[B37] Yavari F. Jamil A. Mosayebi S. M. Vidor L. P. Nitsche M. A. (2018). Basic and functional effects of transcranial Electrical Stimulation (tES)-An introduction. *Neurosci. Biobehav. Rev.* 85 81–92. 10.1016/j.neubiorev.2017.06.015 28688701

[B38] Yin D. Luo Y. Song F. Xu D. Peterson B. S. Sun L. (2013). Functional reorganization associated with outcome in hand function after stroke revealed by regional homogeneity. *Neuroradiology* 55 761–770. 10.1007/s00234-013-1146-9 23417103

[B39] Yoon B. W. Morillo C. A. Cechetto D. F. Hachinski V. (1997). Cerebral hemispheric lateralization in cardiac autonomic control. *Arch. Neurol.* 54 741–744. 10.1001/archneur.1997.00550180055012 9193209

[B40] Zaaimi B. Edgley S. A. Soteropoulos D. S. Baker S. N. (2012). Changes in descending motor pathway connectivity after corticospinal tract lesion in macaque monkey. *Brain* 135 2277–2289. 10.1093/brain/aws115 22581799 PMC3381720

[B41] Zuk N. J. Bertrand D. (2019). Neural coding and perception of auditory motion direction based on interaural time differences. *J. Neurophysiol.* 122 1821–1842. 10.1152/jn.00081.2019 31461376 PMC6843087

